# Harnessing nitrate over ammonium to sustain soil health during monocropping

**DOI:** 10.3389/fpls.2023.1190929

**Published:** 2023-07-17

**Authors:** Linxing Zhu, Aichen Liang, Rongfeng Wang, Yaman Shi, Jia Li, RuiRui Wang, Min Wang, Shiwei Guo

**Affiliations:** Jiangsu Provincial Key Lab for Organic Solid Waste Utilization, National Engineering Research Center for Organic-based Fertilizers, Jiangsu Collaborative Innovation Center for Solid Organic Waste Resource Utilization, Nanjing Agricultural University, Nanjing, China

**Keywords:** cucumber, monocropping, nitrogen forms, soil health, plant productivity

## Abstract

**Introduction:**

In achieving food security and sustainable agricultural development, improving and maintaining soil health is considered as a key driving factor. The improvement based on different forms of nitrogen fertilization has aroused great public interest in improving and restoring monocropping obstacles for specific soil problems.

**Methods:**

For this, a short-term cucumber cropping field experiment was conducted in the subtropical region of China under four fertilization treatments: ammonium (AN), nitrate (NN), ammonium with dicyandiamide (AN+DCD), nitrate with dicyandiamide (NN+DCD). In this study, we measured the effects of nitrogen forms addition on plant productivity and soil health in a monocropping system over seven seasons.

**Results:**

To systematically evaluate soil health, a wide range of soil environmental factors were measured and incorporated into the soil health index (SHI) by entropy method. Compared with ammonium treatment (SHI_AN_ = 0.059, SHI_AN+DCD_ = 0.081), the positive effect of nitrate was mainly reflected in improving soil health (SHI_NN_ = 0.097, SHI_NN+DCD_ = 0.094), which was positively correlated with the increase in plant productivity of cucumber after seven seasons of monocropping. The most critical factor affecting SHI is soil ammonium nitrogen content, which was negatively correlated with plant productivity.

**Discussion:**

Nitrate promotes soil health and plant productivity by optimizing soil environmental factors. The study thus emphasized the necessity of nitrate input for the sustenance of soil-crop ecosystems, with the consequent possibility of application of the results in planning monoculture obstacle prevention and management measures.

## Introduction

As the foundation of sustainable agricultural development, soil health plays a pivotal role in ensuring food security and maintaining the functioning of terrestrial ecosystems (Yang T et al., 2020). Environmental, economic, and social benefits such as healthy food and beautiful landscapes require a steady energy supply from healthy soils. In China, more than 95% of cultivated land soil resources have suffered from monocropping obstacles by the end of 2013 (Yang T et al., 2020). As the main culprit of unsustainable agriculture, monocropping not only causes severe land nutrient depletion and accelerates soil degradation, but also makes the single agricultural ecosystem more vulnerable to the threat of pests and diseases. Previous studies revealed that continuous cropping of watermelon, banana, ginseng, and potato increased the abundance of pathogenic microbes (Shen et al., 2018; Gao et al., 2019; Zhang et al., 2020; Gu et al., 2022). In addition, microorganisms involved with nutrient element cycling and the decomposition of harmful allopathic substances, such as *Pseudomonas* spp. and *Bacillus* spp., showed an inhibitory effect (Huang et al., 2019; Luo et al., 2019). Hence, cultivated land protection and management measures are proposed by researchers, including planting systems and fertilization systems.

Fertilizer plays an indispensable role in the soil health and ecosystem function, and it is a critical limiting factor for plant productivity (Baer et al., 2003). Over the past decade, substantial addition of atmospheric nitrogen deposition has been observed by Jia et al. (2014). However, the response of soil health to increase nitrogen levels is not always clear and consistent. The view that a large amount of nitrogen addition damages soil health has been repeatedly proposed to support the argument against fertilization, while other studies have demonstrated otherwise (Treseder, 2008; Zhang et al., 2018). A reasonable fertilization system is an important way to maintain soil health. According to the needs of crops, balanced nitrogen fertilizer application promoted the enhancement of soil health, such as increased plant litter and root biomass and accumulated soil organic matter and microbial biomass. Most studies have examined the effect of the nitrogen application rate on soil health, overlooking on the nitrogen forms (ammonium and nitrate).

Ammonium and nitrate are the main inorganic nitrogen forms absorbed by plants, playing a crucial role in the growth and development of plants. It is important to study the effects of two forms of nitrogen (ammonium and nitrate) on soil properties to support the prediction of improved ecosystems under increased nitrogen deposition. Several studies have reported that microbes and plants preferentially absorb ammonium, as ammonium is more energy-advantageous than nitrate, and the energy costs are lower (Zhang et al., 2017). Another essential feature of ammonium is that it leads to rhizosphere acidification compared to nitrate (Treseder, 2008; Zhang et al., 2017). More specifically, H^+^ offsetting excessive cation uptake by roots was proved to account for the decrease in rhizosphere pH during ammonium fertilization. Hence, many plants are directly inhibited when they absorb ammonium alone due to its special action. In brief, the principal indications of ammonium toxicity include the plant’s overall growth yield, significant leaf chlorosis, decreased root-to-shoot ratio, yield reduction, and even mortality (Britto and Kronzucker, 2002; Li et al., 2010).

Ammonium and nitrate can easily produce a variable effect on soil microbial community biomass excitation and decomposition (Tao et al., 2018; Jia et al., 2019). For instance, the respiration of peat substrate increased with ammonium, but did not change with nitrate (Currey et al., 2009). However, from laboratory incubation trials, the researchers concluded that nitrogen inhibited soil microbial respiration regardless of the form of applied nitrogen based on the results observed (Ramirez et al., 2010; Yang et al., 2022). Based on a long-term forest experiment, ammonium significantly reduces the organic carbon content of the organic layer. In contrast, nitrate affects both the organic carbon content and density fractions of the mineral layer (Geng et al., 2021). Such phenomenon might be more closely related to more inputs from plant sources, rather than to a slower breakdown of organic matter. However, another study suggested that the promoting effect of ammonium on soil organic carbon accumulation is significantly higher than that of nitrate, likely as a result of their different soil pH and microbial enzyme activity (Lu et al., 2011). In the past, there have been a large number of studies on the effects of nitrogen forms on the soil single properties (Treseder, 2008; Zhong et al., 2017). However, there remains a knowledge gap on how nitrogen forms impact comprehensive indexes of soil health, which hampers the efforts to predict soil health in the context of global changes.

To better understand the association between nitrogen forms and soil health in monocropping, we investigated the impacts of ammonium and nitrate on the composition of the microbial community, soil resources (such as nutrient retention and availability), overall soil health, and plant productivity (related to element accumulation and yield). Understanding those complex relationships, two main hypotheses were addressed: (1) soil resources and microbial community properties vary with nitrogen form, and each contributes distinctively to soil health; (2) nitrate would boost the soil health by enhancing the soil resources (e.g., soil enzyme activity) and environmental allocation (e.g., pH and EC) and thereby plant productivity.

## Materials and methods

### Experiment design and sampling

This trial was set up at Changzhou city, Jiangsu Province, China (31°27′ N, 119°19′ E) in 2017 for the monocropping of spring and autumn cucumber (Jin Chun 4). The trials in the spring and autumn seasons were initiated in early March and late July, respectively. The area has a typical subtropical monsoon climate, with an average annual precipitation of 1,033 mm and an average annual temperature of 15.5°C. At the beginning of the experiment, the soil properties were pH 6.8, 25.2 g kg^−1^ organic matter, 1.5 g kg^−1^ total nitrogen, and 40.3 mg kg^−1^ and 182 mg kg^−1^ of available P and K, respectively. The 12 sampling plots (6 m × 2.5 m each) were designed within the greenhouse and assigned to four treatments: ammonium fertilizer (AN), nitrate fertilizer (NN), ammonium fertilizer with dicyandiamide (AN+DCD), and nitrate fertilizer with dicyandiamide (NN+DCD), with three replicates arranged in a completely randomized block design. Dicyandiamide is implemented as a nitrification suppressant in soil.

**Figure 1:**
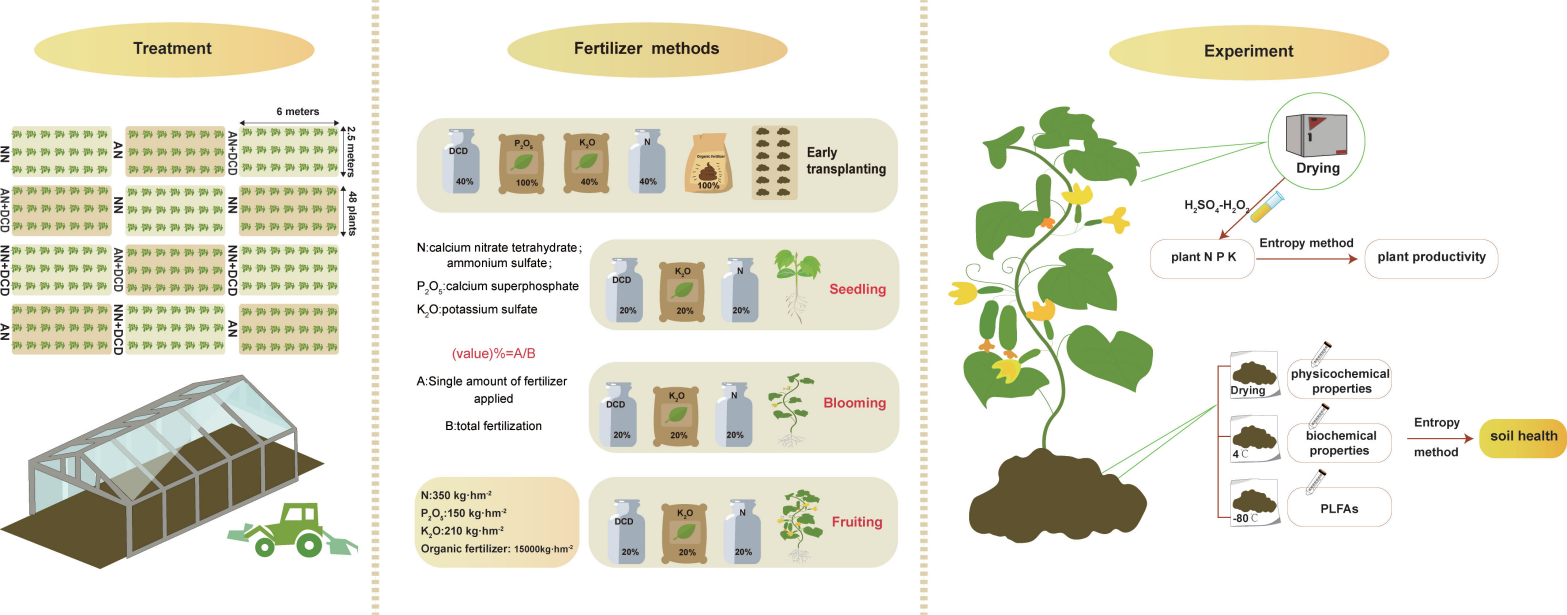
The concept map shows the key experimental arrangements in the current study, including experimental treatment, fertilization methods, and experimental processes. The application of DCD, nitrogen fertilizer, and potassium fertilizer was done four times: 40% of the fertilizer amount was used for applying base fertilizer while the remaining 60% was used for top dressing. Phosphate fertilizer and organic fertilizer were applied once as the base fertilizer.

The chemical fertilizer was applied at the rates of 350 kg N hm^−2^, 150 kg P_2_O_5_ hm^−2^, 210 kg K_2_O hm^−2^, and 10.5 kg DCD hm^−2^ to a depth of 0–20 cm. Calcium nitrate (17.07% N) and ammonium sulfate (21.21% N) were used as sources of nitrate and ammonium, respectively. The P and K fertilizers were prepared from calcium superphosphate (12% P_2_O_5_) and potassium sulfate (34% K_2_O), respectively. Chicken manure containing 31.7% water, 1.3% N, 2.9% P_2_O_5_, and 1.3% K_2_O was applied as an organic fertilizer at the rate of 15,000 kg hm^−2^. Additionally, all of the fertilizer application rates were used for a single growing season (**Figure 1**).

Samples of three cucumber plants selected randomly from each plot were harvested at maturity, and dissected into leaves, stems, and fruits. These components of cucumber were washed with deionized water and oven dried to a constant weight at 70°C to determine the dry-matter biomass. Moreover, dry samples are ground into powder for the analysis of nitrogen, phosphorus, and potassium.

The sustainability yield index (SYI) was used to describe the fluctuating crop yield under different fertilization regimes. A system’s sustainability is increased when its SYI is higher (Reddy and Babu, 2003). The formula is as follows:


$$\text{SYI}=\frac{\text{x}-\text{σ}}{Y_{max}}$$

In the formula, σ (kg hm^−2^) is the standard deviation; *x* (kg hm^−2^) is the average annual yield of the same fertilization treatment in spring; *Y*_max_ (kg hm^−2^) is the maximum yield of in all treatments in spring.

Ten soil subsamples were taken from the cultivated layer (0–20 cm depth) during the cucumber harvest in June 2020 and mixed as a composite sample per plot. For each soil samples, three subsamples were taken: one subsample was dried and stored until physicochemical properties are tested, one subsample was stored at 4°C for biochemical analysis, and the remaining subsample was frozen at −80°C for PLFA analyses.

### Plant nutrient assays

Dried plant samples were digested with H_2_SO_4_-H_2_O_2_ at 260–270°C (Li et al., 2022), after which N concentration was measured using an Auto Analyzer 3 digital colorimeter (BRAN + Lu-EBBE) (Guo et al., 2007). The determination of phosphorus (P) in the digested solution was carried out by the molybdenum-blue method (Xu et al., 2020). The determination of potassium (K) in the digested solution was conducted using a flame photometer (Xu et al., 2019).

### Soil properties assays

Soil pH and EC were determined with a soil-to-water ratio of 1:2.5 by a pH meter (FE28-Meter) and a conductivity meter (DDSJ-308F), respectively. Soil total nitrogen (TN) and organic matter (SOC) were determined using an Elemental Analyzer (Vario MAX; Elementar, Germany). Soil mineral nitrogen (nitrate and ammonium) (NO_3_^−^ and NH_4_^+^) was extracted with 0.01 M CaCl_2_ and was quantified by Bran + Luebbe GmbH-AutoAnalyzer 3 (Norderstedt, Germany), while soil available P (AP) was extracted using NaHCO_3_ and then measured by the molybdenum-blue method. Soil available K (AK) was extracted with NH_4_OAc and determined using a flame photometer.

### Soil extracellular enzyme activities and respiration assays

Urease was determined by the method of May and Douglas (1976). Soil samples were treated with toluene for 15 min and then incubated with urea in citrate buffer at 37°C for 24 h. After filtration, sodium phenol and sodium hypochlorite were added. After 20 min, the color was determined by spectrophotometer at 578 nm. Acid phosphatase was determined according to the description of Sun et al. (2017). The mixed solution of 0.2 ml of toluene, 4 ml of buffer solution, and 1 ml of a disodium p-nitrophenyl phosphate solution was added with 1 g of soil, and then incubated at 37°C for 1 h. The catalase activity was determined by ultraviolet spectrophotometry (Yang R et al., 2020). Hydrogen peroxide solution was added to the soil as a substrate, and saturated aluminum potassium jarosite was quickly added after sealing oscillation for 20 min, and quickly filtered into a triangular flask containing sulfuric acid. The filtrate was subjected to colorimetric analysis using a quartz cuvette at 240 nm. Finally, the sucrase activity was determined by the 5-dinitrosalicylic acid colorimetric method (Wang et al., 2020). The sample was treated with toluene and incubated in water at 37°C for 15 min. Then, sucrose solution and phosphate buffer were added and cultured at 37°C for 24 h. The filtrate was removed after filtration and DNS reagent was added to a boiling water bath for 5 min. After cooling and constant volume, the sample was colorimetric at 540 nm. An enzyme activity unit of urease and invertase was defined by the production of 1 μg of NH_3_-N and 1 mg of reducing sugar per gram of soil sample within 24 h. Catalase was expressed as milligram of hydrogen peroxide decomposed per gram of soil within 20 min. The results of acid phosphatase were expressed as μg of pNP g^−1^ of dry soil h^−1^.

The CO_2_ evolution from the samples that had been precultured at 25°C for 24 h in darkness was measured by a gas chromatograph and then incubated at 25°C in tight containers for 6 h, to evaluate soil basal respiration (Hofman et al., 2003). Soil metabolic entropy (qCO_2_) was calculated as the ratio between soil basal respiration and soil microbial biomass carbon.

### Soil microbial assays

The soil microbial biomass carbon (MBC) and microbial biomass nitrogen (MBN) were quantified using the chloroform fumigation extraction and a 0.5 M K_2_SO_4_ solution extraction method. To calculate MBC or MBN contents, the difference in soil soluble carbon or nitrogen between unfumigated and fumigated samples was determined (Gregorich and Kachanoski, 1991). Soil soluble carbon in the extracts was quantified using a TOC analyzer (Elementar, Germany), and soil soluble nitrogen was detected using the Kjeldahl method. Conversion factors of 0.45 (KE_C_) and 0.54 (KE_N_) were used to calculate average MBC and MBN values (Brookes et al., 1985; Wu et al., 1990).

The composition of the soil microbial community was evaluated by PLFA analysis according to Frostegård and Bååth (1996). Qualitative and quantitative PLFA analysis was performed by GC-MS (a gas chromatograph combined with a mass spectrometer, Thermo Scientific, Bremen, Germany). The PLFAs i14:0, i15:0, a15:0, i16:0, i17:0, and a17:0 were used as indicators of Gram-positive bacteria (G^+^); 16:1ω7c, 17:1ω8c, 18:1ω7c, cy17:0, and cy19:0 were used as indicators of Gram-negative bacteria (G^–^); the PLFAs 10Me16:0, 10Me17:0, and 10Me18:0 were used as indicators of actinomycetes; the PLFAs 16:1ω5c and 18:2ω6,9c were used as indicators of fungi. The total bacterial PLFAs are represented by the combined count of G^+^ PLFAs and G^–^ PLFAs. The PLFAs for soil microbes were expressed as nmol g^−1^ dry soil.

### Soil health index

The soil health index is calculated using the entropy method (Geng et al., 2018; Wu et al., 2018). According to the attribute of the index *j* (e.g., TN and SOC), it is divided into three categories: positive (except EC, qCO_2_, and pH), negative (EC and qCO_2_), and moderate (pH). Among them, the greater the index value, the better the role of the index, which is a positive index (*X*). The smaller the index value, the better the effect of the index. It is a negative index. The moderate index means that when the value of the index is in the middle, the index performs best. Before the soil health index evaluation of the monocropping of cucumber, all the selected evaluation indexes were uniformly processed to unify them into positive index (*X*). The formula is as follows:

#### Reverse indicator transformation

If the index *j* is a negative index, it can be transformed into a positive index (*X*).


$$\text{X}=\frac{1}{\text{j}}$$

#### Moderate indicator conversion

If the index *j* is a moderate index, it can be transformed into a positive index (*X*) by the following formula:


$$\text{X}=\{\begin{array}{c} \frac{2*\left(\text{j}-\text{m}\right)}{\text{M}-\text{m}},\;\;\;\;\;\;\text{m}\leq \;\text{j}<\frac{\text{m}+\text{M}}{2}\\ \frac{2*\left(\text{M}-\text{j}\right)}{\text{M}-\text{m}},\;\;\;\;\;\frac{\text{m}+\text{M}}{2}\leq \text{j}\leq \text{M} \end{array}$$

where *M* and *m* are the maximum and minimum values in the range of index *j*, respectively.

#### Data standardization processing (*X^`^_ij_*)

Different evaluation index units are changeable. All indexes are dimensionless before calculation, which makes the data comparable. The extreme value method is used for dimensionless treatment of indicators, and all the indicators are transformed into interval of 0–1. Then, *X^`^_ij_* was calculated as below:


$${\text{X}}_{\text{i}\text{j}}^{`}=\frac{{\text{X}}_{\text{i}\text{j}}-{\text{m}}_{\text{j}}}{{\text{M}}_{\text{j}}-{\text{m}}_{\text{j}}}$$

where *X_ij_* is the sample *i* of the index *j*, and *m_j_* and *M_j_* are the minimum and maximum values among all the evaluation objects under the same index, respectively.

#### Weighting

1. Calculate the contribution of the sample *i* of the index *j*. In order to make the data operation meaningful, it is necessary to eliminate the zero and negative values. Dimensionless data are translated as a whole, namely, *X^`^_ij_* = *X^`^_ij_* +*α*. At the same time, in order to maximize the retention of the original data, α takes the minimum value closest to *X^`^_ij_*, and *α* = 0.0001.

2. Calculate the characteristic weight of the *i*th evaluated object under the *j*th index:


$${\text{p}}_{\text{i}\text{j}}=\frac{X_{ij}^{`}}{\sum_{i=1}^{n}\text{X}{`}_{ij}}\;,\;\;\;\text{n}=1,2,3,\ldots 12$$

where *p_ij_* is the proportion of the standard value of the sample *i* of the index *j*, and *n* is the number of samples involved in the calculation.

3. Calculate the entropy value *e_j_* of the *j*th index, with the expression:


$${\text{e}}_{\text{j}}=-\frac{1}{\text{l}\text{n}\;\text{n}}\sum_{\text{i}=1}^{\text{n}}{\text{p}}_{\text{i}\text{j}}\text{l}\text{n}\left({\text{p}}_{\text{i}\text{j}}\right),\;\;\;\text{n}=1,2,3,\ldots 12$$

4. Calculate the difference coefficient (*g_j_*):


$${\text{g}}_{\text{j}}=1-{\text{e}}_{\text{j}}$$

5. Determine the weight of the evaluation index:


$${\text{W}}_{\text{j}}=\frac{{\text{g}}_{\text{j}}}{\sum_{\text{j}=1}^{\text{z}}{\text{g}}_{\text{j}}},\;\;\;\;\;\;\text{j}=1,2,3\ldots \;\text{z}$$

where *W_j_* is the importance of the index *j* among all indicators, and *z* refers to the total number of indexes that participate in the calculation.

#### Calculation of soil health (*S*)


$$\text{S}=\sum_{\text{j}=1}^{\text{z}}{\text{w}}_{\text{j}}*{\text{p}}_{\text{i}\text{j}}\;\;,\;\;\;\;\;\;\text{j}=1,2,3\ldots \;\text{z}$$

### Statistical analysis

Data were compared using one-way analysis of variance (ANOVA) at the end of each bioassay in the IBM SPSS 19.0 software program (SPSS Inc., USA). The ggcor package in R was used to test the correlation between soil physicochemical and biological properties, and its relationship with the plant quality index. Based on the Bray–Curtis distance matrix, the effects of nitrogen forms on microbial composition were analyzed by principal coordinate analysis (PCoA). Permutational multivariate analysis of variance (PEROMOVA with 999 permutations) was executed by utilizing the “adoni” function within the “vegan” R package. Causal relationships between soil properties and plant productivity were tested by partial least squares path modeling (PLS-PM) using “plspm”. Statistical significance was deemed to be achieved when the alpha level was equal to or less than 0.05.

## Results

### Plant productivity

**Figure 2:**
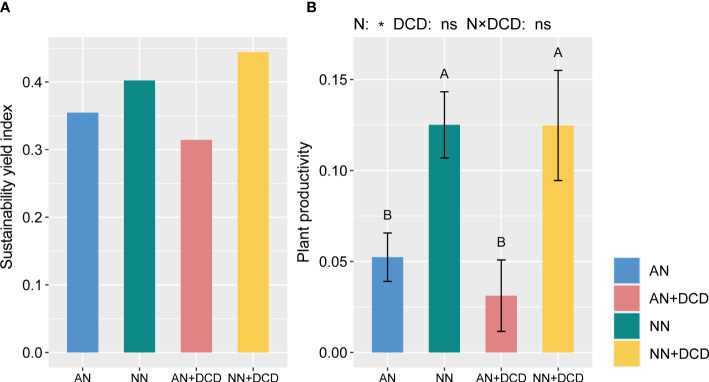
Sustainability yield index **(A)** and plant productivity **(B)** under different fertilization treatments. Data points represent the means and standard deviations of three replicates. Different capital letters denote significant differences (*p*< 0.05) with Duncan’s multiple range test. The plant productivity is calculated in the same way as the soil health index, which includes aboveground biomass, yield, and nutrient accumulation. AN, ammonium fertilizer; NN, nitrate fertilizer; AN+DCD, ammonium fertilizer with dicyandiamide; NN+DCD, nitrate fertilizer with dicyandiamide. N, Nitrogen form; *p< 0.05; ns, *p* > 0.05.

After seven seasons, all of the plant productivity-related parameters were significantly affected by nitrogen forms. Overall, the productivity of plants significantly increased by approximately 140% on nitrate treatment (NN) compared to ammonium treatment (AN) (**Figure 2**). Additionally, in terms of nutrient accumulation (**Figure S1**), nitrate treatment (NN) provides more nutrients to the plant, whether it is nitrogen, phosphorus, or potassium. Likewise, we observed a similar pattern in the DCD treatment, wherein plant productivity was significantly higher with nitrate treatment (NN+DCD) than with ammonium treatment (AN+DCD). Furthermore, the present results revealed that neither DCD nor the interactions of nitrogen forms and DCD could affect the plant productivity-related parameters (**Figure S1**).

### Soil physicochemical and biological properties

The physicochemical properties of the soil were significantly influenced by the fertilization regime, with the exception of TN and SOC (**Table 1**). Different nitrogen forms directly affected the nitrate-to-ammonium ratio (NO_3_^−^/NH_4_^+^), and nitrate treatment (NN) was significantly higher than the ammonium treatment (AN). All the treatments were acidic in pH, but ammonium treatment (AN, pH = 4.14) had lower acidity than nitrate treatment (NN, pH = 5.99). However, soil AP (131 mg kg^−1^) and AK (346 mg kg^−1^) with ammonium treatment (AN) added were both elevated. With respect to EC, application of nitrate treatment (NN) significantly decreased the electrical conductivity (EC) by approximately 150 μS cm^−1^ compared to ammonium treatment (AN). Overall, the addition of DCD did not alter the trend of physicochemical properties between the nitrate and ammonium nitrogen treatments. Nevertheless, our findings revealed that DCD application caused a notable increase in soil pH while decreasing soil EC (AN vs. AN+DCD or NN vs. NN+DCD).

**Table 1:** Effect of different forms of nitrogen on soil physical and chemical properties.

Treatment	TN (g kg^−1^)	SOC (g kg^−1^)	NH_4_^+^ (mg kg^−1^)	NO_3_^−^ (mg kg^−1^)	NO_3_^−^/NH_4_^+^	AP (mg kg^−1^)	AK (mg kg^−1^)	pH	EC (μS cm^−1^)
AN	1.67 ± 0.07 a	16.8 ± 0.58 a	29.2 ± 4.63 a	119 ± 7.69 b	4.15 ± 0.59 b	131 ± 1 6.8 a	346 ± 5.29 a	4.14 ± 0.15 c	2,500 ± 166 a
NN	1.65 ± 0.09 a	17.1 ± 0.94 a	2.24 ± 0.52 b	208 ± 1.68 a	96.2 ± 20.5 a	93.4 ± 18.0 b	255 ± 27.7 bc	5.99 ± 0.11 a	2,026 ± 89.5 b
AN+DCD	1.64 ± 0.08 a	17.1 ± 0.71 a	37.7 ± 8.29 a	120 ± 42.1 b	3.23 ± 0.98 b	115 ± 18.6 ab	332 ± 32.6 ab	4.55 ± 0.23 b	2,269 ± 55.0 ab
NN+DCD	1.61 ± 0.11 a	17.1 ± 0.76 a	3.25 ± 1.77 b	235 ± 30.9 a	96.8 ± 68.1 a	88.3 ± 13.9 b	264 ± 79.3 c	6.09 ± 0.16 a	1,549 ± 155 c

TN, soil total nitrogen; SOC, soil organic carbon; NH_4_^+^, soil ammonium nitrogen; NO_3_^−^, soil nitrate nitrogen; AP, soil available phosphorus; AK, soil available potassium; EC, soil electrical conductivity. Values are means of three replicates ± standard deviations. Means followed by the same letter for a given factor are not significantly different (p< 0.05; Duncan test). AN, ammonium fertilizer; NN, nitrate fertilizer; AN+DCD, ammonium fertilizer with dicyandiamide; NN+DCD, nitrate fertilizer with dicyandiamide.

Soil effective nutrient content, biological activity, and energy circulation are mainly dominated by soil microorganisms. Soil microbial nutrient content differed notably under different fertilization treatments. Nitrate (NN)-treated MBC increased by 150 mg kg^−1^ compared with ammonium treatment (AN) (**Table 2**). Unexpectedly, for MBN content, the administration of nitrogen in different forms was ineffective, and the difference between treatments was not significant. It was noted that similar to MBC, both the ratio of MBN and TN (MBN/TN) and the ratio of MBC and SOC (MBC/SOC) were prominently impacted by fertilization regimes. Compared with ammonium treatment (AN), both MBN/TN and MBC/SOC were significantly increased (by 50%) in nitrate treatment (NN). Basal respiration in soil was determined, and there were no significant differences in different nitrogen forms. In contrast, the soil metabolic entropy (qCO_2_) of nitrate (NN) was significantly lower than that of ammonium treatment (AN) (**Table 2**), by approximately 50%.

**Table 2:** Effects of different nitrogen forms on soil microbial biomass.

Treatment	MBC (mg·kg^−1^)	MBN (mg·kg^−1^)	MBC/SOC	MBN/TN	SBR (μg·g^−1^·h^−1^)	qCO_2_
AN	198 ± 79.6 c	80.2 ± 21.6 b	0.01± 0.00 b	0.05 ± 0.01 b	3.22 ± 0.23 a	18.4 ± 8.41 a
NN	384 ± 20.5 b	114 ± 10.9 a	0.02 ± 0.00 a	0.07 ± 0.00 a	3.53 ± 0.39 a	9.18 ± 0.66 b
AN+DCD	278 ± 44.0 c	102 ± 10.9 ab	0.02 ± 0.00 b	0.06 ± 0.00 ab	3.51 ± 0.21 a	12.8 ± 1.72 ab
NN+DCD	490 ± 26.5 a	125 ± 20.0 a	0.03 ± 0.00 a	0.08 ± 0.01 a	3.69 ± 0.51 a	7.55 ± 1.33 b

MBC, soil microbial biomass carbon; MBN, soil microbial biomass nitrogen; SBR, soil base respiration; qCO_2_, soil metabolic entropy. Values are means of three replicates ± standard deviations. Means followed by the same letter for a given factor are not significantly different (p< 0.05; Duncan test). AN, ammonium fertilizer; NN, nitrate fertilizer; AN+DCD, ammonium fertilizer with dicyandiamide; NN+DCD, nitrate fertilizer with dicyandiamide.

**Figure 3:**
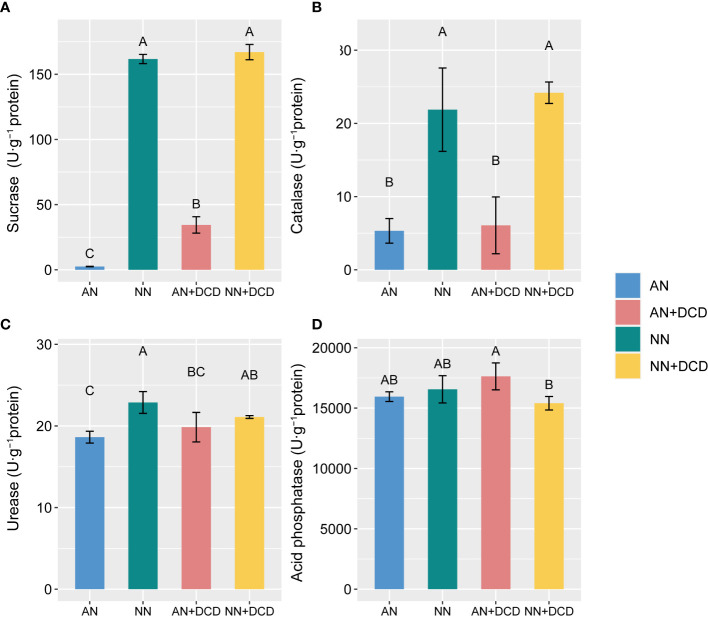
Response of soil enzyme activity to different forms of nitrogen after monocropping for the seven seasons. **(A)** Sucrase; **(B)** catalase; **(C)** urease; **(D)** acid phosphatase. Data points represent the means and standard deviations of three replicates. Different capital letters denote significant differences (*p*< 0.05) with Duncan’s multiple range test. AN, ammonium fertilizer; NN, nitrate fertilizer; AN+DCD, ammonium fertilizer with dicyandiamide; NN+DCD, nitrate fertilizer with dicyandiamide.

Nutrient and redox-related microbial enzyme activities were directly affected by soil nitrogen forms. There was an overall increase in soil enzyme activity upon nitrate addition. Specifically, in soils (**Figure 3**), nitrate treatment (NN) obviously increased the activities of sucrase, catalase, and urease. In contrast, significant reductions in acidic phosphatase activity were observed with the nitrate treatment, as compared with the ammonium treatment (AN). Furthermore, regarding soil biological properties, we observed that DCD supplementation had no significant impact, except for the notable increase in MBC (AN vs. AN+DCD or NN vs. NN+DCD) and sucrase (AN vs. AN+DCD).

### Microbial community structure

Soil microbial community composition (based on PLFAs) varied with fertilization regimes, with PC1 explaining 49.67% of the variation and PC2 explaining 28.79% (**Figure S2A**). Across all fertilization treatments (**Table 3**), the total amount of PLFAs in soil hardly changed, with bacterial PLFA concentration being the highest (76%–82%), followed by fungi (17%–24%) and actinomycetes (1%). The concentrations of bacteria and actinomycete PLFAs did not differ significantly among the treatments. However, ammonium treatment (AN) increases soil fungal biomass, which is more pronounced in DCD treatment. At the same time, nitrate treatment (NN) decreased the ratio of G^+^ to G^–^ (G^+^/G^–^) and increased the ratio of bacteria to fungi (B/F) in rhizosphere soil, compared with ammonium treatment (AN). In line with the majority of other soil properties, our results revealed no significant effect arising from the addition of DCD on soil microorganisms.

**Table 3:** Effects of different nitrogen forms on soil PLFAs.

Treatment	Total PLFAs (nmol·g^–1^)	Bacterial PLFAs (nmol·g^–1^)				Actinomycetic PLFAs (nmol·g^–1^)	Fungal PLFAs (nmol·g^–1^)	B/F
		Total PLFAs	G^+^ PLFAs	G^−^ PLFAs	G^+^/G^−^			
AN	21.2 ± 2.46 a	16.6 ± 2.23 a	10.8 ± 1.62 a	5.80 ± 0.65 ab	1.85 ± 0.13 b	0.21 ± 0.02 a	4.41 ± 0.34 ab	3.76 ± 0.41 ab
NN	20.5 ± 2.02 a	16.6 ± 2.12 a	9.55 ± 0.87 a	7.07 ± 1.41 a	1.37 ± 0.20 c	0.17 ± 0.03 a	3.73 ± 0.47 bc	4.51 ± 0.90 ab
AN+DCD	21.2 ± 3.75 a	16.0 ± 3.54 a	10.9 ± 2.31 a	5.04 ± 1.24 b	2.18 ± 0.09 a	0.22 ± 0.09 a	4.99 ± 0.14 a	3.19 ± 0.64 b
NN+DCD	17.8 ± 2.39 a	14.6 ± 1.57 a	9.34 ± 1.36 a	5.26 ± 0.30 ab	1.77 ± 0.20 b	0.15 ± 0.04 a	3.08 ± 0.83 c	4.88 ± 0.79 a

G^+^ PLFAs, soil Gram-positive bacterial; G^−^ PLFAs, soil Gram-negative bacteria; B/F, the ratio of bacteria to fungi; G^+^/G^−^, the ratio of Gram-positive bacteria to Gram-negative bacteria. Values are means of three replicates ± standard deviations. Means followed by the same letter for a given factor are not significantly different (p< 0.05; Duncan test). AN, ammonium fertilizer; NN, nitrate fertilizer; AN+DCD, ammonium fertilizer with dicyandiamide; NN+DCD, nitrate fertilizer with dicyandiamide.

**Figure 4:**
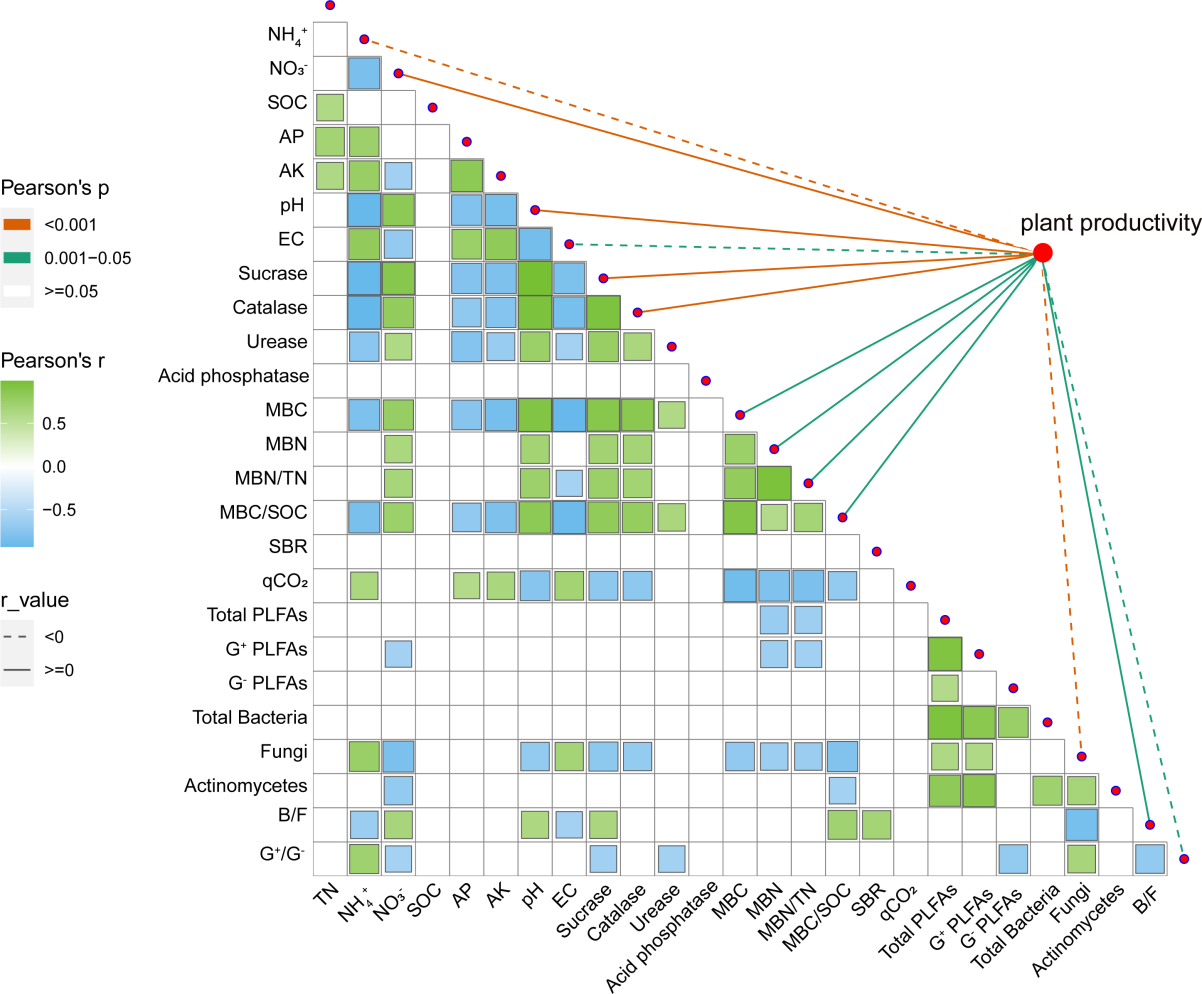
Correlation between soil physicochemical properties and biological properties, and the relationship between various factors and plant productivity. SOC, soil organic carbon (g kg^−1^); TN, soil total nitrogen (g kg^−1^); AP, soil available phosphorus (mg kg^−1^); AP, soil available potassium (mg kg^−1^); NO_3_^−^, soil nitrate nitrogen (mg kg^−1^); NH_4_^+^, soil ammonium nitrogen (mg kg^−1^); EC, soil electrical conductivity(μS cm^−1^); MBC, soil microbial biomass carbon content (mg kg^−1^); MBN, soil microbial biomass nitrogen content (mg kg^−1^); MBN/TN, the ratio of soil microbial biomass nitrogen content to total nitrogen; MBC/SOC, the ratio of soil microbial biomass carbon content to soil organic carbon; SBR, soil basal respiration (μg g^−1^ h^−1^); qCO_2_, soil metabolic entropy; G^+^ PLFAs, Gram-positive bacteria (nmol g^–1^); G^–^ PLFAs, Gram-negative bacteria (nmol g^–1^); B/F, the ratio of bacteria to fungi; G^+^/G^–^, the ratio of Gram-positive bacteria to Gram-negative bacteria. All correlation analysis use Pearson’s correlation. Correlation coefficient *r* value is shown in the figure.

As a visual analysis, we mapped a heat map (**Figure 4**) of the correlation between the physicochemical and biological properties of the soil and calculated the correlation coefficients. **Figure 4** showed a significant negative correlation of soil NH_4_^+^, AP, AK, and EC with soil enzyme activity, MBC, and MBC/SOC, and a significant positive correlation with soil respiration entropy. In addition, NH_4_^+^ and EC were negatively correlated with the ratio of fungi to bacteria, and NH_4_^+^ was positively correlated with the fungi and G^+^/G^–^ alone. Regarding the correlation with other soil properties, soil NO_3_^−^ and NH_4_^+^ exhibited opposite trends. The soil’s NO_3_^−^ is positively correlated with MBN and MBN/TN, and there is no significant relationship between pH and G^+^/G^–^. Although the physical and chemical properties have a greater impact on biological properties, the importance of the total amount of microorganisms cannot be ignored. Total PLFAs, G^+^, and soil fungi were significantly and negatively correlated with MBN and MBN/TN. In addition, MBC and MBC/SOC were also negatively correlated with fungi.

### Quantitative examination of the effects of fertilization

**Figure 5:**
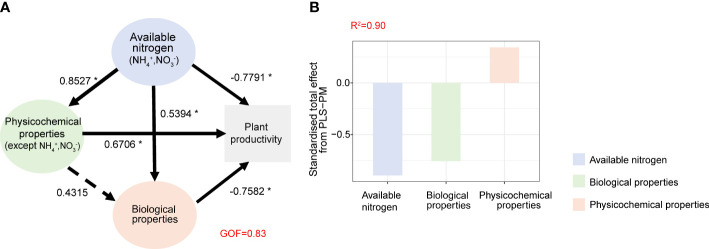
The partial least squares path models (PLS-PM) illustrating the direct and indirect effects of available nitrogen, physical–chemical properties (except NH_4_^+^, NO_3_^−^) and biological properties on cucumber plant productivity **(A)** and standardized total effects on plant health from PLS-PM **(B)**. Numbers on the arrowed lines indicate normalized path coefficient. The dotted arrows represent non-significant path relationships. The GOF index represents the goodness of fit. Asterisks represent significant effects: *p< 0.05; **p< 0.01; ***p< 0.001.

The partial least squares path model was used to reveal the possible ways of nitrogen addition affecting plant productivity (**Figure 5**). This analysis provides the most appropriate exponential model based on our data (GOF = 0.83). Our findings indicate that available nitrogen (NH_4_^+^ and NO_3_^−^) and biological properties significantly and negatively influenced plant productivity (−0.89 and −0.75, respectively), whereas soil physicochemical properties (except NH_4_^+^ and NO_3_^−^) had a significant positive impact (0.34) (**Figure 5B**). Together, these factors explained 90% (*R* ^2 =^ 0.9) of the variation in plant productivity of cucumbers (**Figure 5B**).

**Figure 6:**
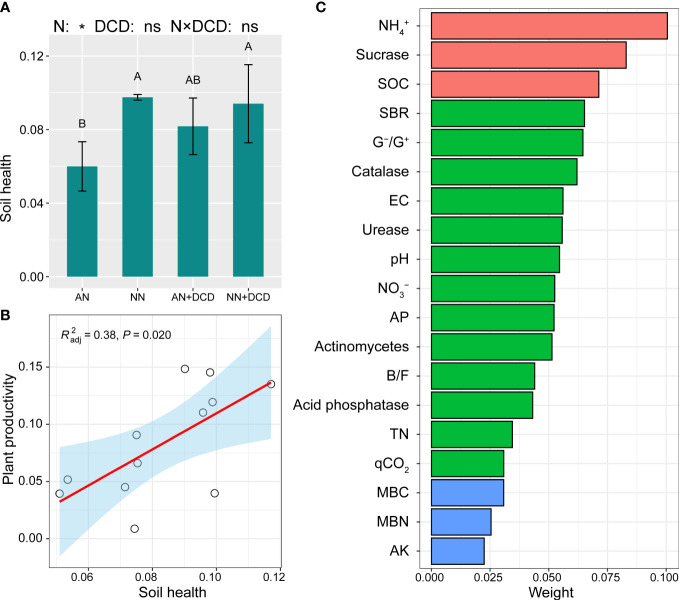
The entropy method determined the response of soil health index **(A)** and plant productivity to different nitrogen forms **(B)**, and calculated the weight **(C)** of soil physicochemical and biological indexes in soil health. SOC, soil organic carbon (g kg^−1^); TN, soil total nitrogen (g kg^−1^); AP, soil available phosphorus (mg kg^−1^); AK, soil available potassium (mg kg^−1^); NO_3_^−^, soil nitrate nitrogen (mg kg^−1^); NH_4_^+^, soil ammonium nitrogen (mg kg^−1^); EC, soil electrical conductivity (μS cm^−1^); MBC, soil microbial biomass carbon content (mg kg^−1^); MBN, soil microbial biomass nitrogen content (mg kg^−1^); MBN/TN, the ratio of soil microbial biomass nitrogen content to total nitrogen; SBR, soil basal respiration (μg g^−1^ h^−1^); qCO_2_, soil metabolic entropy; B/F, the ratio of bacteria to fungi; G^–^/G^+^, the ratio of Gram-negative bacteria to Gram-positive bacteria. Data points represent the means and standard deviations of three replicates. Different capital letters denote significant differences (*p*< 0.05) with Duncan’s multiple range test. AN, ammonium fertilizer; NN, nitrate fertilizer; AN+DCD, ammonium fertilizer with dicyandiamide; NN+DCD, nitrate fertilizer with dicyandiamide. N, Nitrogen form; *p< 0.05; ns, *p* > 0.05.

Further analysis of soil factors and plant productivity showed that NH_4_^+^, EC, fungi, and G^+^/G^–^ were significantly negatively correlated with plant productivity (**Figure 4**). Soil nitrate nitrogen, pH, sucrase, catalase, MBC, MBN, MBN/TN, MBC/SOC, and the ratio of bacteria and fungi (B/F) were significantly positively correlated (**Figure 4**). It also has the same trend for a single plant productivity index (such as yield, biomass, etc.) (**Figure S2B**). The comprehensive score of soil health was calculated by entropy method. As shown in **Figure 6C**, in the soil health evaluation system, NH_4_^+^ and sucrase have higher weight (0.100 and 0.083, respectively) while MBC, MBN, and AK have smaller weight (0.031, 0.025, and 0.0225, respectively). The comprehensive score of soil health treated with nitrate treatment (NN and NN+DCD) was significantly higher than ammonium treatment (AN and AN+DCD), and the specific ranking from high to low was NN+DCD (0.098), NN (0.094), AN+DCD (0.082), and AN (0.060) (**Figure 6A**). Nevertheless, no discernible impact of DCD addition on soil health was observed in our study (AN vs. AN+DCD or NN vs. NN+DCD) (**Figure 6A**). Finally, we performed a correlation analysis between the comprehensive score of soil health and plant productivity, which showed a positive correlation between these two factors (**Figure 6B**, **Figure S2C**).

## Discussion

### Nitrate increased plant productivity

The physiological metabolic processes of plants are controlled by nitrogen, such as nitrogen metabolism, photosynthesis, respiration, and absorption of mineral elements, which determine the different growth effects of plants. Since nitrate addition increased plant productivity over time in our test site, compared to ammonium addition (**Figure 2**, **Figure S1**), this, to some extent, resembles previous findings indicating that the absorption of K^+^ is reduced with the rise of NH_4_^+^ concentration in watermelon, foxtail algae, and tobacco, respectively, when ammonium serves as the exclusive source of nitrogen. This inhibition is probably caused by the ions’ competition taking place within nonselective cation channels and potassium-specific channels (Lu et al., 2015). Furthermore, our previous findings showed that nitrate treatment promoted the increase of root length, which might also be one of the reasons for the weak nutrient absorption capacity of ammonium treatment (**Figure 2**, **Figure S1**) (Wang et al., 2016).

Interestingly, the sustainability yield index under nitrate treatment (NN and NN+DCD) was higher than that of ammonium treatment (AN and AN+DCD) (**Figure 2**). This phenomenon could be explained by the fact that nitrate is more conducive to maintaining the stability of microbial flora, thereby alleviating monocropping obstacles (Gu et al., 2020). Thus, it is reasonable to speculate that the effect of different forms of nitrogen on monoculture obstacles is not a simple factor, but a combination of multiple factors, such as soil health, and plant characteristics. Unexpectedly, in this study, the effects of dicyandiamide on cucumber yield and nutrient uptake were not signified under different nitrogen forms. **Table 4** (PERMANOVA) shows that variations in plant productivity, soil microbial community, and soil nutrients were all best explained (*R* ^2^) by nitrogen form (73.3%, 34.5%, and 63.7%, respectively) and, to a lesser extent, by DCD (0.9%, 11.9%, and 12.7%, respectively) and the interaction between nitrogen form and DCD (0.3%, 9.12%, and 2.79%, respectively). This unknown phenomenon is likely to be affected by soil temperature, soil texture, microbial activity, and other factors, which needs further study (Guiraud and Marol, 1992; Grundmann et al., 1995).

**Table 4:** Results from PERMANOVA testing the effects of nitrogen versus DCD on plant productivity, soil nutrients, and soil microbial community.

	Df	Pseudo-*F*	*p*-value	*R* ^2^ (%)	Pseudo-*F*	*p*-value	*R* ^2^ (%)	Pseudo-*F*	*p*-value	*R* ^2^ (%)
		Plant productivity			Soil microbial community			Soil nutrients		
Nitrogen form	1	23.0	0.003	73.3	6.23	0.001	34.5	31.5	0.001	67.3
DCD	1	0.280	0.638	0.9	2.13	0.126	11.9	5.97	0.036	12.7
Nitrogen form × DCD	8	0.111	0.839	0.3	1.64	0.187	9.12	1.30	0.249	2.79
Residuals	11			25.5			44.4			17.1

### Optimizing soil physicochemical properties by nitrate

It is generally believed that the NH_4_^+^ is easily converted to NO_3_^−^ when NH_4_^+^ is used as the substrate for nitrification under oxygen-rich conditions. In contrast, the high ammonium (29.2 mg kg^−1^, 37.7 mg kg^−1^) that still exists under ammonium treatment (AN and AN+DCD) in our study was presumably attributed to the large reduction in nitrification rate under ammonium treatment (AN and AN+DCD) by the low pH (**Table 1**). Other studies observed that the rate of nitrification increased with the increase in pH, when the pH value was 3.7–8.6. This could be due to pH that increases the growth of nitrifying microorganisms in soil (Jiang et al., 2015). On the other hand, according to previous reports, another important reason for low pH inhibition of nitrification rate is the direct inhibition of nitrification enzyme activity (Jia et al., 2014). Notably, characteristics of ion release from roots during the nutrient uptake period of plants under ammonium treatment (AN and AN+DCD) might expose more H^+^, capable of reducing soil pH (Telles-Pupulin et al., 1996; Evans et al., 2008; Ghorbani et al., 2008). In addition, we found a response to different nitrogen forms by root exudates of cucumber, which may eventually result in citric acid accumulation in ammonium treatment (Wang et al., 2016). Accordingly, the changes in carbon and nitrogen metabolism in plants are regarded as one of the reasons for the regulation of soil pH when exposed to different nitrogen forms. The results showed that the soil pH was more stable during nitrate treatment (NN and NN+DCD) and that there was a significant positive correlation between pH and plant productivity in all treatments (**Figure 4**, **Table 1**). These findings indicated that nitrate treatment (NN and NN+DCD) effectively reduced soil acidification and promoted soil health, thus creating a suitable environment for monocrop growth. The study findings displayed no notable influence on soil total nitrogen and organic matter among treatments. This observation could possibly be associated with the shorter duration of continuous cropping. However, significant deviations in soil available potassium and phosphorus were visible among treatments. These variations probably arose from the growth differences of cucumbers under different nitrogen forms. Notably, the nitrate nitrogen treatment expedited cucumber growth and yield formation, leading to greater nutrient absorption from the soil during the crop harvest stage.

### Nitrate improves soil biological properties

Understanding the mechanisms of soil microbial carbon and nitrogen content, and how they respond to nutrient enrichment, is essential for accurate predictions and management of ecosystem health and related functions. Given that the result of our study is not in line with earlier reports on the significant positive correlation between soil organic carbon and microbial carbon, it is also therefore likely that carbon components are the dominant drivers of this relationship (**Figure 4**). We speculated that this phenomenon was caused by higher active carbon components produced by nitrate treatment (NN and NN+DCD). Previous research, based on preferential utilization of unstable active carbon sources by soil microorganisms, has also highlighted the potential importance of roots and aboveground parts of plants in unstable carbon source turnover through litter production (Wang and Wang, 2006; Geng et al., 2021). Therefore, our results, together with previous studies, emphasized the objectivity of higher microbial biomass carbon and nitrogen under nitrate treatment (NN and NN+DCD) (**Table 2**). In fact, soil available nitrogen supply potential and organic carbon turnover rate were controlled by MBN/TN and MBC/SOC, respectively. The soils treated with nitrate (NN and NN+DCD), especially those fertilized with DCD, showed higher magnitudes of MBC/SOC and MBN/TN compared to the soils treated with ammonium (AN and AN+DCD). This suggests that microbial immobilization under nitrate treatment (NN and NN+DCD) is beneficial for the enrichment of soil nutrient sources, which provides indispensable support for soil health (**Table 2**).

After further analysis (**Figure 3**), we found that carbon-gaining enzymes and nitrogen-gaining enzymes showed high activity in nitrate treatment (NN and NN+DCD), most likely due to an elevated strong unstable carbon input ability by nitrate additions, given that rich energy sources stimulate microbial secretion of soil enzymes related to carbon and nitrogen cycling (Paz-Ferreiro et al., 2011). The inhibitory effect of ammonium treatment (AN and AN+DCD) on soil enzyme activity may be more closely related to abiotic factors (soil pH and NH_4_^+^) than biotic factors (microbial biomass). On the other hand, the authors believe that this is partly due to the direct toxicity of NH_4_^+^ and acidic soil to microorganisms and enzymes (Luo et al., 2016). Consistent with our results, NH_4_^+^ and pH were negatively and positively correlated with soil enzyme activities, respectively (**Figure 4**). In addition, it is well known that the increase of catalase is conducive to soil remediation of monocropping. Therefore, under monocropping conditions, long-term application of nitrate can make the soil more mature, result in less toxic substances, and better maintain soil health. However, soil acid phosphatase activity was higher in AN+DCD, which is probably related to lower pH and higher fungal PLFAs in AN+DCD.

Regardless of nitrogen forms, exogenous nitrogen input might not cause changes in the total number of soil microorganisms, at the current deposition rate, because microbes living in high nitrogen soils can adapt to nitrogen-rich environments (**Table 3**). Here, it does not mean that nitrogen forms have no significant effect on soil microbial community composition at other levels (such as species composition) (**Figure S2A**). After ammonium application, we found that soil microbial community changed from “bacteria” to “fungi”, and fungi were negatively *(p< 0.01)* correlated with the overall plant productivity (**Figure 4** and **Table 3**). We speculate that the decline in plant productivity under ammonium treatment (AN and AN+DCD) was caused by fungal accumulation. This is in line with earlier reports on the directional transformation of rhizosphere microorganisms in monocropping crops (Wei et al., 2017). In a similar study, ammonium input changed the microbial community structure with fungal biomass experiencing greater change than bacterial biomass, resulting in a decreased ratio of B to F and a more saprophytic fungal dominance of microbial populations (Geng et al., 2021). In fact, other studies also reported that nitrate addition reduced soil fungal biomass by gradually inhibiting the growth of white rot fungi or the activity of ligninase (Pregitzer et al., 2008; Frey et al., 2014). Otherwise, the evaluation of root exudates and soil microorganisms showed that the diversity of root exudates (heterogeneity of soil microbial resources) was also an important factor driving the composition of the soil microbial community.

With regard to the ratio of G^+^ to G^–^, there was a widely held assumption that it can be used to characterize soil nutrient status. G^–^ had been grown rapidly in nutrient-rich soil relative to other nutrient-poor soil, which was opposite to G^+^. Results from our study showed that nitrate amendment decreased the ratio of G^+^ to G^–^ (**Table 3**), consistent with previous studies on the subtropical field soil, and indicating that the nutritional stress of nitrate treatment (NN and NN+DCD) was weaker than that of ammonium treatment (AN and AN+DCD) (Kramer and Gerd, 2008), which directly affected plant productivity. The qCO_2_ value reflects the intensity of soil microbial respiration and, hence, serves as a direct measure of the microbial community’s stability (Anderson and Domsch, 2010). Nitrate treatment (NN and NN+DCD) showed a significant reduction in qCO_2_ levels (**Table 2**), indicating its strong potential in preserving the soil microbial community and high substrate utilization rate. Finally, monocropping drove soil microorganisms to an unfavorable environmental state, but nitrate gave soil microorganisms a stronger ability to resist disturbance and maintain stability, thus providing quality assurance for soil health.

### Healthy soil promotes crop production

A long-term positive correlation between soil health and field plant productivity has been reported in grain crop systems (Nunes et al., 2018). This is similar to the result in **Figure 6B**, where there was a significant positive correlation between soil health and aboveground productivity (*R* ^2 =^ 0.38, *p* = 0.02). As shown in **Figure 6**, the comprehensive score of soil treated with nitrate treatment (NN and NN+DCD) was higher (NN = 0.097 and NN+DCD = 0.094), which was related to the richer nutrient content and higher biological activity of soil treated with nitrate treatment (NN and NN+DCD). In addition, it may be related to the direct effect of ammonium on soil degradation. pH, soil enzyme activity, and microbial biomass significantly decreased with the addition of ammonium nitrogen. Therefore, this study supports the second hypothesis that nitrate fertilization is one of the important ways to promote soil sustainable function by improving soil health.

## Conclusions

**Figure 7:**
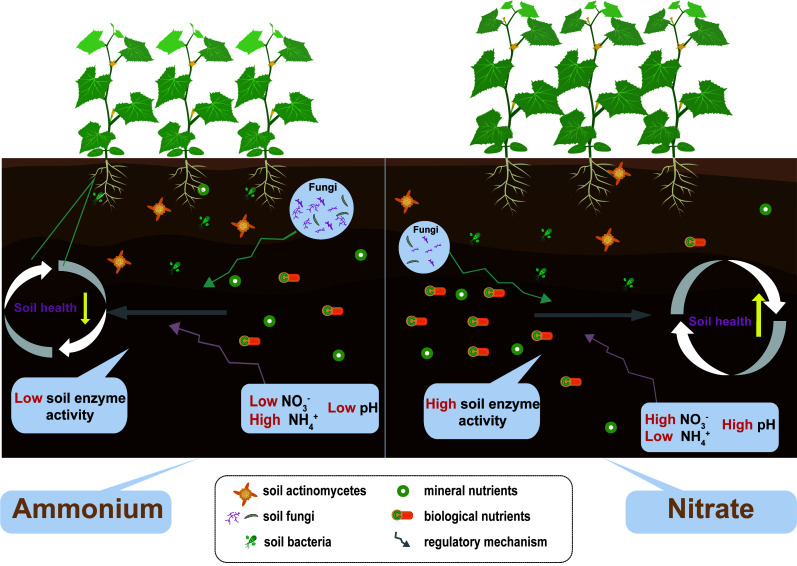
The concept map shows the effects of different nitrogen forms on soil health and plant productivity. The yellow vertical arrows correspond to the soil health and soil stability change intensity. Plant size corresponds to plant productivity.

Nitrate fertilization is more conducive to the absorption and utilization of nutrients, yields formation of monocropping, and delays soil acidification and salinization (**Figure 7**). Furthermore, nitrate fertilization helps to maintain the nutrient structure of soil microbial communities, increases their stability, and promotes soil nutrient transformation and utilization, leading to an improvement in soil health. Hence, nitrate fertilizer application is preferred in the monocropping system of cucumbers. The current findings advance the knowledge of different forms of nitrogen fertilization effects on plant productivity and its role in shaping soil health. Overall, we suggest that agricultural sustainable management by integrating amendments of nitrogen form could enhance regulation of soil ecosystem processes, sustaining the synergies between soil health and crop productivity.

## Data availability statement

The original contributions presented in the study are included in the article/**Supplementary Material**. Further inquiries can be directed to the corresponding author.

## Author contributions

MW conceived the ideas and designed the study; LZ and AL were used in field and laboratory experiments, respectively. LZ analyzed the data and wrote the manuscript with substantial contributions from RFW, YS, JL, RRW, SG, and MW. All authors contributed to the article and approved the submitted version.
