# Supplementary material for: Harnessing nitrate over ammonium to sustain soil health during monocropping
Source: Front Plant Sci. 2023 Jul 17;14:1190929. doi: 10.3389/fpls.2023.1190929 (PMC10389047; doi:10.3389/fpls.2023.1190929)
Supplement: Supplementary file 1 [file DataSheet_1.docx]

**Harnessing nitrate to sustain soil health during** **monocropping**

Linxing Zhu, Aichen Liang, Rongfeng Wang, Yaman Shi, Jia Li, RuiRui Wang, Min Wang*, Shiwei Guo

Jiangsu Provincial Key Lab for Organic Solid Waste Utilization, National Engineering Research Center for Organic-based Fertilizers, Jiangsu Collaborative Innovation Center for Solid Organic Waste Resource Utilization, Nanjing Agricultural University, Nanjing, 210095, China.

*Corresponding author: Min Wang

*E-mail address*: minwang@njau.edu.cn;

Tel.: +86-25-8439-5212

**Supplementary Information includes:**

**Figures S1 to S4**

***Supplementary Figures:***


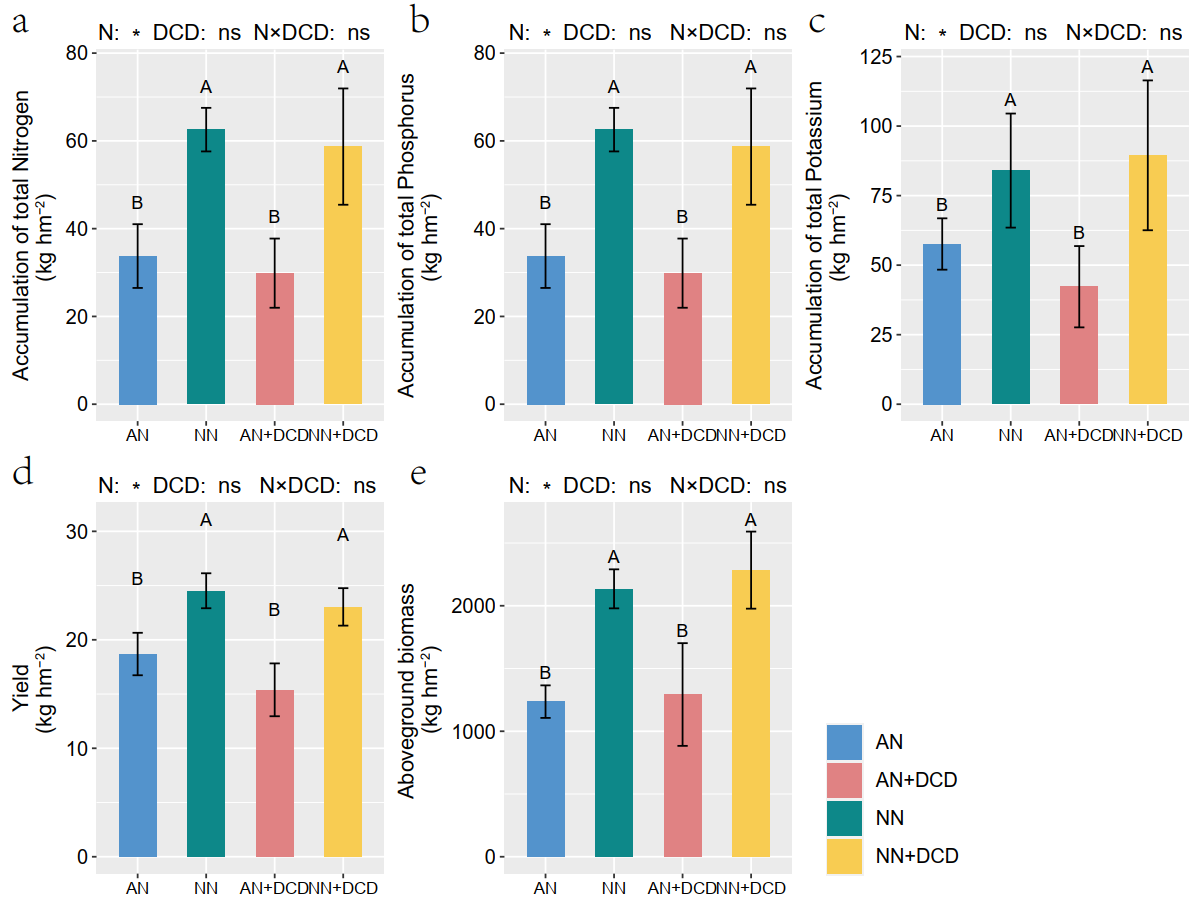


**Fig.S1** Plant productivity of cucumber after monocropping in the seventh season. (a)The accumulation of total nitrogen of cucumber, (b) The accumulation of total phosphorus cucumber, (c) The accumulation of total potassium, (d) cucumber yield, (e) aboveground biomass under different fertilization treatments. Data points represent the means and standard deviations of three replicates. Different capital letters denote significant differences (*P<0.05*) with Duncan’s multiple range test. AN, ammonium fertilizer, NN, nitrate fertilizer, AN+DCD, ammonium fertilizer with dicyandiamide, NN+DCD, nitrate fertilizer with dicyandiamide; N, Nitrogen form; *, *P<0.05*; ns, *P>0.05*.


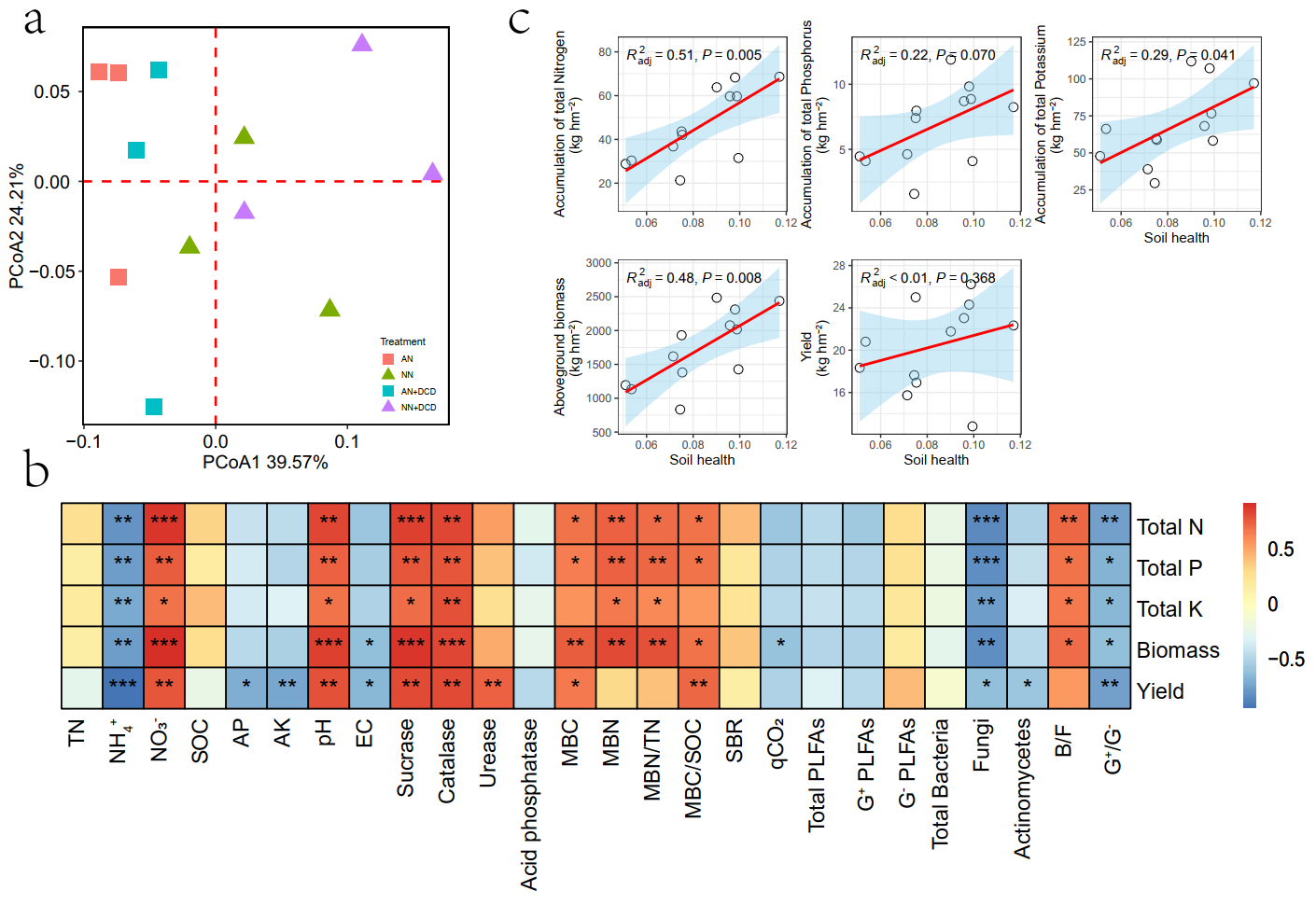


**Fig.S2** (a) Constrained principal coordinate analysis of the PLFAs from cucumber soil. (b) Correlation between soil properties and plant productivity after seven consecutive cropping seasons. (c)Correlation analysis between soil health and plant productivity. SOC, soil organic carbon (g kg^-1^); TN, soil total nitrogen (g kg^-1^); AP, soil available phosphorus (mg kg^-1^); AP, soil available potassium (mg kg^-1^); NO_3_^-^, soil nitrate nitrogen (mg kg^-1^); NH_4_^+^, soil ammonium nitrogen (mg kg^-1^); EC, soil electrical conductivity(μS∙cm^-1^); MBC, soil microbial biomass carbon content (mg kg^-1^); MBN, soil microbial biomass nitrogen content (mg kg^-1^); MBN/TN, the ratio of soil microbial biomass nitrogen content to total nitrogen; MBC/SOC, the ratio of soil microbial biomass carbon content to soil organic carbon ; SBR, soil basal respiration (μg∙g^-1^∙h^-1^); qCO_2_, soil metabolic entropy; G^+^ PLFAs, soil gram-positive bacteria (nmol·g^–1^); G^-^ PLFAs, soil gram-negative bacteria (nmol·g^–1^);B/F, the ratio of bacteria to fungi; G^+^/G^-^, the ratio of gram-positive bacteria to gram-negative bacteria; Total N, accumulation of total nitrogen (kg hm^-2^); Total P, accumulation of total phosphorus (kg hm^-2^); Total K, accumulation of total potassium (kg hm^-2^). All correlation analysis use Pearson’s correlation. Correlation coefficient r value is shown in the figure, * *P < 0.0*5, * * *P < 0.01*, * * * *P < 0.001*.AN, ammonium fertilizer, NN, nitrate fertilizer, AN+DCD, ammonium fertilizer with dicyandiamide, NN+DCD, nitrate fertilizer with dicyandiamide.

***Supplementary Tables:***

**Table S1** Dimensionless processing results of soil health index values of different fertilization treatments

|  | AN1 | AN2 | AN3 | NN1 | NN2 | NN3 | AN+DCD1 | AN+DCD2 | AN+DCD3 | NN+DCD1 | NN+DCD2 | NN+DCD3 |
| --- | --- | --- | --- | --- | --- | --- | --- | --- | --- | --- | --- | --- |
| TN | 0.147055 | 0.086102 | 0.06191 | 0.11324 | 0.02782 | 0.128832 | 0.134644 | 0.042783 | 0.070628 | 0.129381 | 1.47E-05 | 0.05759 |
| NH_4_^+^ | 0.142731 | 0.113448 | 0.158669 | 0.007769 | 0.002796 | 0.004326 | 0.205347 | 0.132819 | 0.202266 | 0.01664 | 2.05E-05 | 0.013168 |
| NO_3_^-^ | 0.025156 | 0.02827 | 0.03998 | 0.123205 | 0.121497 | 0.119788 | 0.080288 | 0.014059 | 1.85E-05 | 0.185434 | 0.1308 | 0.131503 |
| SOC | 0.128702 | 0.054462 | 2.03E-05 | 0.059412 | 2.03E-05 | 0.202941 | 0.059412 | 0.044564 | 0.188093 | 0.183144 | 0.024767 | 0.054462 |
| AP | 0.190748 | 0.104623 | 0.138446 | 0.050578 | 1.91E-05 | 0.093117 | 0.158422 | 0.063828 | 0.096255 | 0.074288 | 0.003855 | 0.025821 |
| AK | 0.112459 | 0.117865 | 0.116784 | 0.074616 | 0.049207 | 0.075697 | 0.118405 | 0.084077 | 0.108134 | 0.083266 | 1.18E-05 | 0.059479 |
| pH | 0.084042 | 0.046218 | 2.11E-05 | 0.11343 | 0.074231 | 0.054632 | 0.085437 | 0.211447 | 0.177851 | 0.068629 | 2.11E-05 | 0.084042 |
| EC | 0.045411 | 0.026675 | 0.015974 | 0.09955 | 0.082919 | 0.075028 | 2.13E-05 | 0.088439 | 0.036136 | 0.178111 | 0.212594 | 0.139142 |
| Sucrase | 1.58E-05 | 0.000233 | 2.7E-05 | 0.145842 | 0.149204 | 0.152445 | 0.025361 | 0.036598 | 0.027957 | 0.147779 | 0.157845 | 0.156693 |
| Catalase | 0.023939 | 0.031473 | 0.008871 | 0.117457 | 0.10738 | 0.179331 | 1.79E-05 | 0.053134 | 0.026576 | 0.139588 | 0.153056 | 0.159177 |
| Urease | 1.89E-05 | 0.012322 | 0.045487 | 0.172799 | 0.188847 | 0.107539 | 0.037999 | 0.124656 | 0.013392 | 0.09684 | 0.105399 | 0.0947 |
| Acid phosphatase | 0.037342 | 0.074664 | 0.071227 | 0.045854 | 0.157166 | 0.073191 | 0.147099 | 0.089888 | 0.204309 | 0.048637 | 2.04E-05 | 0.050602 |
| MBC | 0.056497 | 0.040525 | 1.5E-05 | 0.095283 | 0.108838 | 0.096571 | 0.050468 | 0.079982 | 0.054091 | 0.133176 | 0.150431 | 0.134122 |
| MBN | 0.075813 | 0.042432 | 1.57E-05 | 0.107768 | 0.076226 | 0.110978 | 0.096212 | 0.05799 | 0.077101 | 0.108809 | 0.08927 | 0.157384 |
| SBR | 0.082857 | 2.41E-05 | 0.004157 | 0.089148 | 0.172786 | 0.01604 | 0.040335 | 0.101465 | 0.12148 | 0.240621 | 0.051106 | 0.07998 |
| qCO_2_ | 0.055469 | 0.049888 | 1.67E-05 | 0.096015 | 0.093682 | 0.114219 | 0.055271 | 0.077458 | 0.047568 | 0.103879 | 0.166546 | 0.139987 |
| B/F | 0.072904 | 0.035263 | 0.082076 | 0.101005 | 0.167558 | 0.059071 | 1.75E-05 | 0.013592 | 0.073114 | 0.175085 | 0.080284 | 0.14003 |
| G^+^/ G^-^ | 0.045596 | 0.090342 | 0.050414 | 0.185868 | 0.242008 | 0.115656 | 0.011728 | 2.42E-05 | 0.023752 | 0.105153 | 0.035251 | 0.094207 |
| Actinomycetes | 0.112644 | 0.088107 | 0.131422 | 0.048004 | 0.050577 | 0.10319 | 0.047466 | 0.063881 | 0.249031 | 2.49E-05 | 0.09101 | 0.014643 |

Note: SOC, soil organic carbon (g kg^-1^); TN, soil total nitrogen (g kg^-1^); AP, soil available phosphorus (mg kg^-1^); AK, soil available potassium (mg kg^-1^); NO_3_^-^, soil nitrate nitrogen (mg kg^-1^); NH_4_^+^, soil ammonium nitrogen (mg kg^-1^); EC, soil electrical conductivity(μS∙cm^-1^); MBC, soil microbial biomass carbon content (mg kg-1); MBN, soil microbial biomass nitrogen content (mg kg-1); SBR, soil basal respiration (μg∙g^-1^∙h^-1^); qCO_2_, soil metabolic entropy; B/F, the ratio of bacteria to fungi; G^+^/G^-^, the ratio of gram-positive bacteria to gram-negative bacteria.

**Table S2** Entropy value, difference coefficient and weight of evaluation index for soil health

|  | TN | NH_4_^+^ | NO_3_^-^ | SOC | AP | AK | pH | EC | Sucrase | Catalase | Urease | Acid phosphatase | MBC | MBN | SBR | qCO_2_ | B/F | G^+/^ G^-^ | Actinomycetes |
| --- | --- | --- | --- | --- | --- | --- | --- | --- | --- | --- | --- | --- | --- | --- | --- | --- | --- | --- | --- |
| Entropy | 0.924274 | 0.779289 | 0.884701 | 0.843418 | 0.885326 | 0.950574 | 0.88021 | 0.876987 | 0.817908 | 0.863885 | 0.87767 | 0.905236 | 0.932427 | 0.944138 | 0.85681 | 0.932368 | 0.903367 | 0.858282 | 0.887187 |
| Diversity actor | 0.075726 | 0.220711 | 0.115299 | 0.156582 | 0.114674 | 0.049426 | 0.11979 | 0.123013 | 0.182092 | 0.136115 | 0.12233 | 0.094764 | 0.067573 | 0.055862 | 0.14319 | 0.067632 | 0.096633 | 0.141718 | 0.112813 |

Note: SOC, soil organic carbon (g kg^-1^); TN, soil total nitrogen (g kg^-1^); AP, soil available phosphorus (mg kg^-1^); AK, soil available potassium (mg kg^-1^); NO_3_^-^, soil nitrate nitrogen (mg kg^-1^); NH_4_^+^, soil ammonium nitrogen (mg kg^-1^); EC, soil electrical conductivity(μS∙cm^-1^); MBC, soil microbial biomass carbon content (mg kg-1); MBN, soil microbial biomass nitrogen content (mg kg-1); SBR, soil basal respiration (μg∙g^-1^∙h^-1^); qCO_2_, soil metabolic entropy; B/F, the ratio of bacteria to fungi; G^+^/G^-^, the ratio of gram-positive bacteria to gram-negative bacteria.
